# A Comparative Study of MALDI MSI Versus DESI MSI Applied to Questioned Document Examination

**DOI:** 10.1002/rcm.70135

**Published:** 2026-07-09

**Authors:** Veronika Tibljas, Simona Francese, Marjory Da Costa Abreu, Charlotte Lefebvre, Robert Bradshaw

**Affiliations:** ^1^ Centre for Mass Spectrometry Imaging, Biomolecular Sciences Research Centre City Campus, Sheffield Hallam University Sheffield UK; ^2^ School of Computing and Digital Technologies City Campus, Sheffield Hallam University Sheffield UK; ^3^ Organic Synthesis and Mass Spectrometry Laboratory, Research Institute for Biosciences University of Mons Mons Belgium

## Abstract

**Rationale:**

In forensic investigations, questioned document examination (QDE) provides critical information for document authentication and the detection of forgeries. Conventional QDE approaches often require separate analyses of inks and of any potential fingerprints. This study compares two mass spectrometry imaging (MSI) techniques for QDE, enabling the simultaneous analysis of inks and fingerprints deposited onto paper, providing chemical and biometric information from a single sample.

**Methods:**

Two mock forgery samples containing two ballpoint pen inks, printed ink and latent fingerprints were each analysed using either desorption electrospray ionisation mass spectrometry imaging (DESI MSI) or matrix‐assisted laser desorption/ionisation mass spectrometry imaging (MALDI MSI) and the results were compared. Analyses were conducted using state‐of‐the‐art multi reflection time‐of‐flight (MRT) mass spectrometry instrumentation, providing high resolving power and sub‐ppm mass accuracy.

**Results:**

Both techniques successfully differentiated between two blue ballpoint pen inks by detecting ink components present in one pen but absent from the other (despite them being optically indistinguishable). Chemical information associated with the deposited fingerprints was also successfully detected and mapped using both DESI MSI and MALDI MSI. However, MALDI MSI outperformed DESI MSI in terms of the clarity of biometric information obtained.

**Conclusions:**

Overall, the strengths and limitations of DESI MSI and MALDI MSI for QDE were evaluated, demonstrating the potential of each technique for QDE. This assessment helps identify the most suitable approach for the analysis of this type of forensic casework by showing the benefits and reliability of each technique.

## 1 Introduction

Document forgery is a widespread and complex offense that often involves multiple legal authorities, affecting areas such as contractual agreements, property ownership, financial dealings and the integrity of public records [1]. Questioned document examination (QDE) employs a range of methods designed to support and strengthen forensic evidence. These methods may include analysis and comparison of inks to characterise their chemical composition and origin [2], examination of intersecting lines to determine the order of deposition [3], inspection of handwriting characteristics [4], assessment of paper type and fibre composition [5] and fingermark detection [6]. Traditional methods rely mainly on visual examination, microscopy and basic chromatographic techniques, whereas more modern approaches incorporate multispectral and hyperspectral imaging as well as advanced spectroscopic and chemical analyses [7].

Mass spectrometry (MS)–based techniques have been increasingly used within QDE [8] and show great promise for ink analysis, particularly in cases where the conventional methodologies may not provide sufficient information. Since traditional approaches rely primarily on visual interpretation, they are prone to subjectivity and may fail to provide the level of specificity required for objective and scientifically supported differentiation. MS methods address this limitation by enabling molecular identification (ID) of ink components. Common MS techniques that have been employed to analyse various types of inks include time‐of‐flight secondary ion mass spectrometry (TOF‐SIMS) [9], direct analysis in real time–mass spectrometry (DART‐MS) [10], gas chromatography–mass spectrometry (GC‐MS) [11], high performance liquid chromatography–mass spectrometry (HPLC‐MS) [12], ion mobility spectrometry (IMS) [13], desorption electrospray ionisation (DESI MS) [14] and matrix‐assisted laser desorption ionisation (MALDI MS) [15]. Furthermore, mass spectrometry imaging (MSI) capabilities enable in situ differentiation of diverse inks by mapping and identifying their specific chemical components (such as dyes and additives) [16]. Previous work from our group has shown how DESI MSI in negative ion mode was able to detect oleic and linoleic acids, which are commonly found in various ink formulations, whereas MALDI MSI in positive ion mode revealed common ink dyes. Importantly, this in situ analysis of inks on documents is particularly valuable for forensic applications, as it provides spatially resolved chemical information directly from the samples. Furthermore, QDE using MS‐based approaches could reveal hidden intelligence, including fingermarks, thereby potentially offering indications of who may have handled or accessed the document.

Fingermark detection remains a fundamental component in the investigation of many types of crime, including document forgery. The 2022 Fingerprint Visualisation Manual [17] provides foundational guidance for forensic document examiners (FDEs). The process for porous substrates, including paper, typically begins with a visual examination, followed by fluorescence examination under alternate light sources to detect latent marks. Subsequent steps in the development workflow often include the application of chemical reagents (e.g., indandione and ninhydrin). The final stages involve treatment with physical developer and subsequent enhancement techniques [17]. Previous research demonstrates how the application of MS techniques enables chemical characterisation of fingerprint residues in addition to traditional ridge detail visualisation. Bradshaw et al. successfully employed MALDI MSI to fingerprints taken from a real crime scene, even after conventional forensic enhancement techniques have been used [18]. This process, commonly referred to as ‘molecular fingerprinting’, enables the collection of chemical information from fingerprints, typically through MALDI mass spectrometry profiling (MSP) and MSI. When obtained through MSI, the chemical profiles of the fingerprints can be used to visualise ridge and furrow patterns, thereby providing biometric information. Spatial mapping of fingerprint components not only enables the identification of a potential suspect but can also provide insights into their lifestyle. For example, presence of illicit drugs and their metabolites [18, 19], pharmaceuticals [20, 21] and explosive residues [22]. These findings led to molecular fingerprinting being recognised as a Category B technique in 2023 within the UK Home Office and the Defence Science and Technology Laboratory (DSTL) edited Fingermark Visualisation Manual [17]; this recognition acts as a recommendation to adopt this technique in major crime when all other conventional avenues have been exhausted. DESI MSI has previously been applied for the visualisation of latent fingermarks [23, 24, 25]_,_ including those contaminated with condom lubricants [26] or drug residues [27]. Recent work has demonstrated the successful application of DESI MSI to latent fingermarks on paper, even after optical development with Oil Red O (ORO) [28]. However, imaging was limited to selected regions of the marks, and analyte delocalisation was observed for certain lipid species. Indeed, Krishna et al. (2026) [29] investigated these challenges in applying DESI MSI to fingerprint imaging and reported, within the conditions investigated and optimised, the so‐called ‘bleeding effect’, whereby analyte migration from the fingerprint ridges into the furrows affected image quality.

Building on these prior studies, we further investigate and compare the capabilities of both DESI and MALDI MSI for QDE samples containing two types of inks (ballpoint pen and printed) overlapped with a fingerprint thus simulating a scenario that could be encountered in practice. Both techniques were applied using state‐of‐the‐art MRT instruments offering ≥ 200 000 FWHM mass resolution, sub‐ppm mass accuracy and lateral resolution of 15 μm without oversampling [30]. Our previous work [16] has demonstrated that DESI achieves optimal performance for these types of samples in negative ion mode, whereas MALDI is more effective in positive ion mode. Accordingly, the analyses were conducted in the ionisation polarity most appropriate to each technique to ensure optimal analytical performance. To assess the potential of each technique for this type of analysis, the authors examined (a) how effectively each method differentiates between the two blue ballpoint pens; (b) how well each method detects and maps molecular species from a groomed fingerprint; (c) the extent to which the integrity of the sample is preserved during and following analysis and (d) the number of peaks detected for each feature using the different ionisation methods. This study aims to evaluate the forensic opportunities afforded by each method, when applied independently, to support QDE, particularly in cases where conventional methodologies may not have been able to answer the question posed.

## 2 Experimental

### 2.1 Chemicals

Trifluoroacetic acid (TFA) and α‐cyano‐4‐hydroxycinnamic acid (α‐CHCA) were purchased from Sigma‐Aldrich (Poole, UK). Formic acid and HPLC grade acetone, methanol (MeOH) and acetonitrile (ACN) were obtained from Fisher Scientific (Loughborough UK). Double‐sided conductive carbon tape was purchased from TAAB (Aldermaston, UK). Milli‐Q water was obtained from the in‐house system. Analysis involved two blue ballpoint pens (different manufacturers), black ink was printed using a bizhub C659 printer from Konica Minolta (Tokyo, Japan), on a white office paper A4 75 GSM (Woodland Trust, UK).

### 2.2 Instrumentation

MALDI MSI and DESI MSI analyses were conducted on a SELECT SERIES Multi Reflecting Time‐of‐Flight (MRT) instrument (Waters Corporation Wilmslow, UK). Both instruments were run with 0.2‐s scan time, 50‐μm pixel size and raster rate of 250 μm s^−1^. Continuous Lockmass Correction (CLMC) was implemented during the acquisition for both instruments during analysis. For MALDI MSI in positive ion mode, CLMC was applied on the sodiated matrix peak [α‐CHCA + Na]^+^ at *m*/*z* 212.0324 and for DESI MSI in negative ion mode, CLMC was applied on Leu–Enkephalin peak at *m*/*z* 554.2615. The quad profile was set to automatic for data acquisition in a full mass range (*m*/*z* 50–2400). The data acquisition and processing were conducted using MassLynx version 4.2 and HDI version 1.8 (Waters Corporation, Wilmslow, UK), respectively. Images were normalised by the total ion count (TIC) and contrast and intensity were set to maximise the quality of each image. Mean prediction error for the DESI MSI and MALDI MSI analyses, following calibration for both instruments were < 0.2 ppm.

### 2.3 Sample Preparation

Two simulated contracts were produced, each incorporating the standard signature line labelled ‘SIGNATURE’ and a simulated signature using two optically indistinguishable blue ballpoint pens. The signature line and ‘SIGNATURE’ was printed using an in‐house printer *bizhub C659* Konica Minolta (Tokyo, Japan). The simulated signatures were prepared in the same way by writing ‘Leonie’ using two different blue ballpoint pens on white A4 office paper. The ‘Leo’ portion of the signature was written with ballpoint pen 1 (PEN 1), while the forged ‘nie’ portion was written with ballpoint pen 2 (PEN 2), both positioned above the signature line. Groomed fingerprints were prepared by rubbing fingertips across the forehead, nose, and chin five times to collect an abundance of endogenous material, producing a sebum‐rich mark which was subsequently deposited over the ink from both pens in each sample [31]. A full schematic view of the simulated signed document is shown in Figure S1. The two samples were secured to a single glass slide using double sided‐carbon tape prior to analysis. The samples were analysed using MRT mass spectrometers, one in DESI and other in MALDI mode. For DESI MSI analysis, a solvent mixture of 95:5 MeOH:H_2_O spiked with 100 pg μL^−1^ of Leu‐enkephalin was employed, sprayed at a flow rate of 2 μL min^−1^. Both DESI and MALDI MSI parameters were subjected to extensive and systematic optimisation for each of the target features (fingerprints, inks and substrates) prior to analysis. Data acquisition was performed in negative ion mode, with a capillary voltage set at 0.5 kV, cone voltage at 40 V, source temperature of 120°C and the heated transfer line set to 30°C. No additional sample preparation was required for DESI MSI analysis. Prior to MALDI MSI analysis, 5 mg mL^−1^ of α‐CHCA matrix in 70:30 MeOH:0.5% TFA_aq_ was spray‐coated the using HTX M^3+^ Sprayer (HTX Imaging, North Carolina, USA) using a solvent flow rate of 100 μL min^−1^ and a N_2_ pressure of 8 psi, for eight layers, at a velocity of 1300 mm min^−1^.

### 2.4 Data Analysis

Data was processed and visualised using HDI v1.8 (Waters Corporation, Wilmslow, UK) with MS resolution set to 200 000, *m*/*z* window of 0.005 and the number of most intense peaks set to 1000. An automated correlation function available in the HDI software was used, which computes an *R* value (0–1) between a selected reference image and all ion images to assist in identifying spatially correlated features. A feature‐based comparison presented a detailed breakdown of the number of peaks detected for each feature using the different ionisation methods (Table 1). Three regions of interest (ROIs) were exported from areas corresponding to ballpoint pens (PEN 1 and PEN 2), printed ink, and the paper substrate (Figure S2). These ROIs were identical in size (16 pixels) and were selected from identical locations for both imaging techniques to compare performance. Average mass spectra were then exported for each ROI using the multivariate analysis (MVA) tool in HDI v1.8.

**TABLE 1:** Comparison of detected *m*/*z* peaks present in different features (PEN 1, PEN 2, printed ink, fingerprint, substrate) following DESI and MALDI MSI.

Feature	No. *m*/*z* using DESI MSI	No. *m*/*z* using MALDI MSI
Pen 1	23	21
Pen 2	10	10
Printed ink	2	30
Fingerprint	28	44
Substrate (paper)	6	20

## 3 Results

### 3.1 Ink Analysis

**FIGURE 1:**
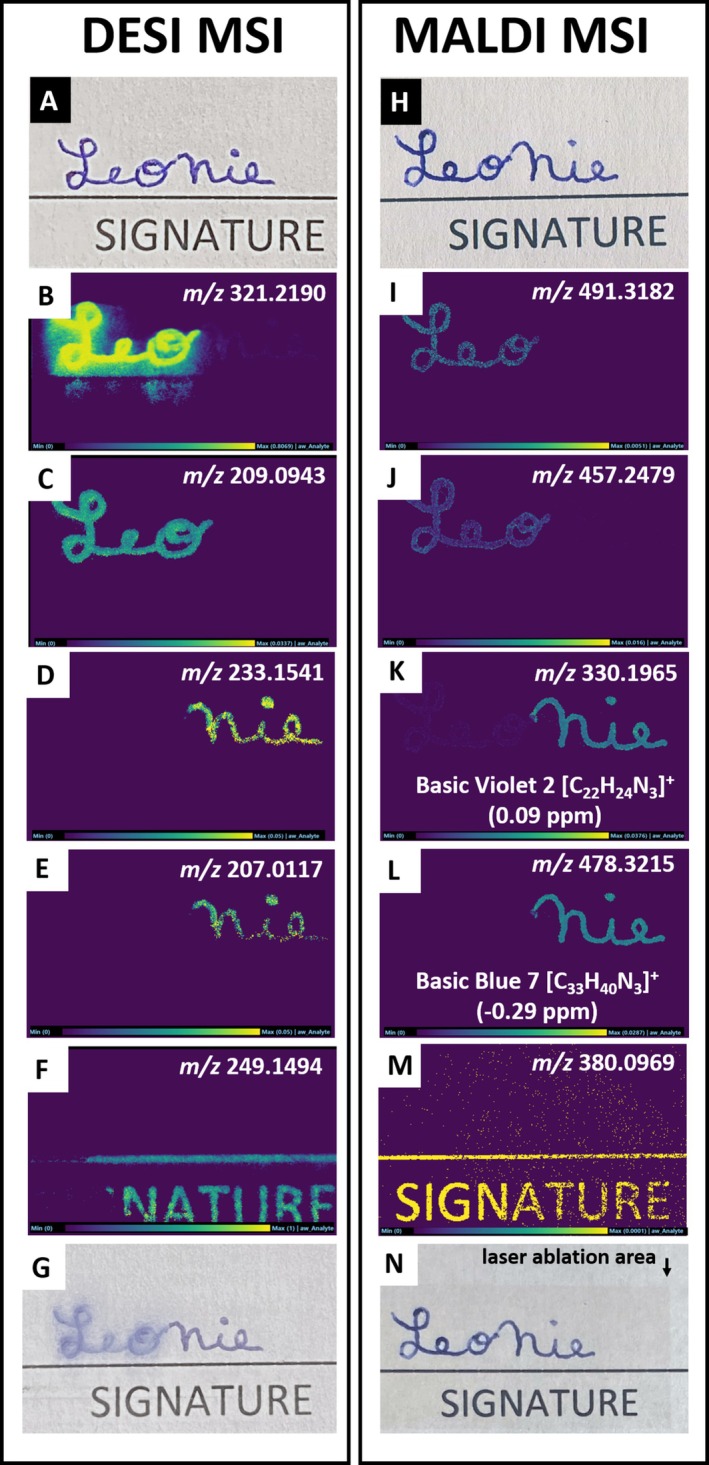
Comparison of DESI and MALDI MSI for QDE. For DESI MSI: Figure 1A is the optical image before analysis, Figure 1B–F shows the ion images, and Figure 1G is the optical image after analysis. For MALDI MSI, Figure 1H is the optical image before analysis, Figure 1I–M present the ion images, and Figure 1N displays the optical image of the sample after analysis.

The ability of DESI and MALDI MSI to assist QDE on samples which had various types of inks are summarised in Figure 1.

### 3.2 Ink Analysis—DESI MSI

As observed in Figure 1, DESI MSI in negative ion mode successfully permitted the recovery of unique ions from both PEN 1 and PEN 2. Specifically, PEN 1 produced ions at *m*/*z* 321.2190 (Figure 1B) and *m*/*z* 209.0943 (Figure 1C), outlining only the first part of the signature (‘Leo’). Some ions within PEN 1 show some delocalisation (Figure 1B), while other ions are unaffected (Figure 1C). This is likely due to the solubility of specific ballpoint pen ink components in the DESI spray solvent, resulting in their migration during the DESI acquisition. Indeed, it has been shown previously that dye‐based colorants can be more readily extracted and soluble in methanol whereas pigment‐based particles, being largely insoluble, remain intact [32]. Furthermore, prior studies suggest that ballpoint pen inks can be categorised as oil‐based, exhibiting higher viscosity and limited paper absorption, or water‐based, which penetrate more deeply into the substrate [33]. PEN 2 ink contained ions at *m*/*z* 233.1541 (Figure 1D) and *m*/*z* 207.0117 (Figure 1E), visualising only the second, forged part of the signature (‘nie’). None of the ions from PEN 2 ink showed delocalisation, indicating that this pen did not contain molecules soluble in the DESI solvent. Consistent with findings in alkyd paint systems [34], solvent effects can also be explained by differences in molecular mobility and changes in the ink matrix. These factors control analyte movement, which helps explain why PEN 1 showed DESI‐induced diffusion while PEN 2 remained spatially stable. Within prior research, DESI MSI demonstrated limited capability in detecting ions from printed inks, likely due to the ink chemistry and its interaction with the paper substrate during printing process [16]. As observed in Figure 1F, an ion at *m*/*z* 249.1494 permitted the visualisation of the printed ink region, though this was limited to areas where a fingerprint was deposited. The authors hypothesise that the ions detected in these regions originate from the fingerprint deposited on top of the printed inks. It is possible that the printed ink acted as a barrier, resulting in fingerprint molecules remaining on the surface, where they are readily ionised. Similar findings were observed in positive ion mode using MALDI MSI, as discussed in subsequent sections of this manuscript.

### 3.3 Ink Analysis—MALDI MSI

For QDE samples, our group had already optimised MALDI MSI in positive ion mode and integrated it into a multimodal workflow [16]. Within the present study, its application as a standalone technique allowed for detection of unique ions from PEN 1 and PEN 2, and from the printed ink. More specifically, PEN 1 showed ions at *m*/*z* 491.3182 (Figure 1I) and *m*/*z* 457.2479 (Figure 1J), permitting the visualisation of only the first part of the signature (‘Leo’). PEN 2 produced ions at *m*/*z* 330.1965 (Figure 1K), putatively identified as Basic Violet 2 [C_22_H_24_N_3_]^+^ (0.09 ppm) and *m*/*z* 478.3215 (Figure 1L) as Basic Blue 7 [C_33_H_40_N_3_]^+^(−0.29 ppm), Selection of these ions visualised only the forged part of the signature (‘nie’). Previous work from our group demonstrated that MALDI MSI outperforms DESI MSI in detecting signals from printed inks [16, 35]. This finding was confirmed in the present study, where MALDI MSI successfully detected signals from the black laser‐printed ink visualising the word ‘SIGNATURE’ and the signature line (Figure 1M). The optical image of the sample following MALDI MSI is shown in Figure 1N, where the ablated area is visible. Interestingly, in contrast to DESI MSI causing delocalisation of some components of PEN1, neither PEN 1 nor PEN 2 showed delocalisation following MALDI MSI, despite the sample being initially sprayed with a matrix solution consisting of 70% methanol. Similarly to the results obtained by DESI MSI, signal enhancement was observed in the printed ink areas overlapped with a fingerprint (Figure S3). Figure S3A–C demonstrates enhancement of ions in the fingerprint regions, indicated by brighter colours in the molecular images. In cases where the ions originate from printed inks, this enhancement could be attributed to improved co‐crystallisation and enhanced positive charge transfer arising from salts present in sweat [36]. Alternatively, in cases where detected ions originate from fingerprint residues, this enhancement may arise from a similar effect reported for DESI MSI, where fingerprint material ‘sits’ on top of the printed ink surface, where they are more readily ionised. Further studies are necessary to confirm and understand the underlying patterns of these interactions. Figure S3D–F demonstrates the absence of signal from the area where a fingerprint was deposited. Indeed, similar loss of signal and suppression effects of fingerprint ridges on detection of ink components have been reported in previous literature. For example, a study by Bright et al. [37] showed how TOF‐SIMS could be used to identify the deposition order of a fingerprint and a ballpoint pen line. In their study, the authors report that the ion suppression of ink‐derived ions arises from matrix effects and variations of secondary ion yield caused by the fingerprint material. In our study, printed inks that were overlapped with fingerprint also suffered ion suppression, which may arise from several factors, including poor matrix crystal formation during spraying, particularly when ink components become trapped beneath fingerprint residues and are therefore not fully solubilised, or from standard ion suppression effect [38]. Importantly, the presence of these spatially dependent ion‐intensity changes indicates that the MSI techniques also have potential for determining the deposition order of inks and fingerprints.

The obtained results show the value of MALDI MSI in assisting QDEs, as it enables recovery of molecules (in many cases dyes, often readily ionised in positive mode) that can provide an additional layer of confidence in differentiation cases [16, 39, 40]. Within this study, MALDI MSI allowed for the detection of ions from all examined ink types (two blue ballpoint pens and a printed ink). Multiple dyes that were found are shared between the two ballpoint pens, indicating compositional similarity. In addition to these, some unique dyes were also identified.

**FIGURE 2:**
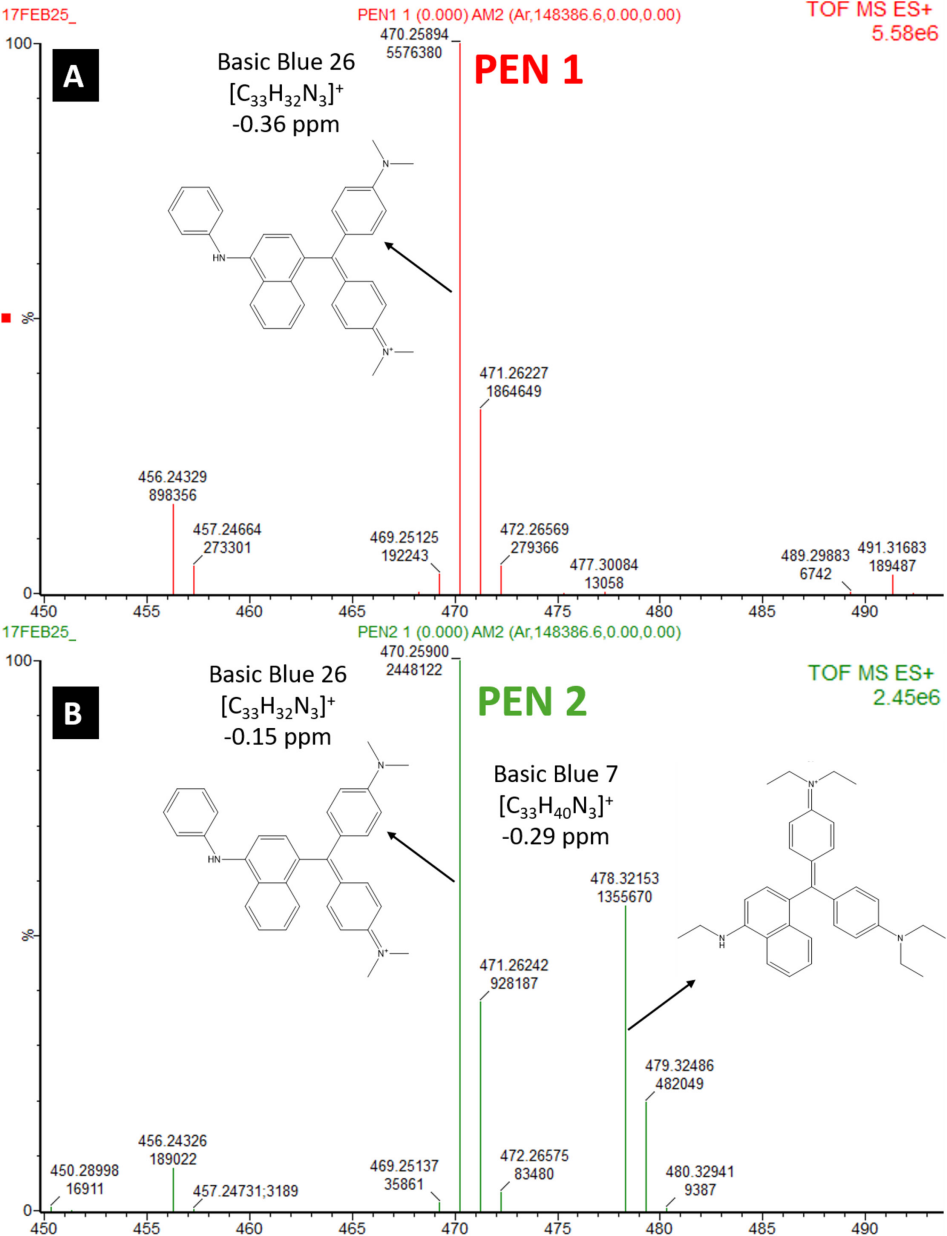
Representative examples of extracted mass spectra from the same size ROIs taken from A: PEN 1 (shown in red) and B: PEN 2 (shown in green). Two ions of interest are shown: *m*/*z* 470.2589 putatively identified as Basic Blue 26 detected in both pens, and ion at *m*/*z* 478.3215 putatively identified as Basic Blue 7 (−0.29 ppm) detected only in PEN 2.

Figure 2 shows extracted MALDI MS spectra from both (A) PEN 1 and (B) PEN 2, revealing distinct chemical profiles and clear differences in the composition of the two pens. The ROIs extracted were identical in size to allow a fair comparison. Both inks share an ion at *m*/*z* 470, putatively identified as Basic Blue 26 (−0.36 ppm in PEN 1 and −0.15 ppm in PEN 2) [41]. The two ballpoint pens exhibited noticeably different signal intensities for the ion representing this dye. Specifically, PEN 1 showed an ion intensity of 5.58 × 10^6^ whereas PEN 2 displayed a much lower value of 2.44 × 10^6^ for the same ion. The variation in the quantity of this dye between the two pens is further supported by the molecular ion image (Figure S4). This could have arisen from several factors, one of which is the unique chemical formulation of each pen that may differ in the types and proportions of dyes used. Indeed, the distinct chemical formulations of the two ballpoint pens is more obvious in the presence of an ion at *m*/*z* 478.32153 putatively identified as Basic Blue 7 (−0.29 ppm) [41], detected in PEN 2 only, and an unknown ion at *m*/*z* 491.31683 present in PEN 1 only.

Despite both being considered ‘soft ionisation’ techniques, the postanalysis optical images of the samples (Figure 1H,O) highlight a clear difference between DESI and MALDI. After MALDI MSI, both the ballpoint pen and printed ink remain clearly visible, with no delocalisation. This could be because in DESI MSI, the directed solvent jet applies a stronger combination of the mechanical force and solvation to the surface to the surface. In contrast, the MALDI MSI matrix spray involves a perpendicular application of matrix as a fine solvent mist which causes less analyte delocalisation. Following MALDI MSI the visible ablation corresponds primarily to removal of the applied matrix crystals rather than substantive removal of the underlying ink material. Notably, the inks remain visually intact following MALDI MSI analysis, however, it is not possible to know the amount of material removed following analysis. Though, by contrast, DESI MSI resulted in visible analyte delocalisation after analysis, which is apparent when comparing the post‐analysis samples and has greater implications for subsequent forensic examination.

### 3.4 Fingerprint Analysis

**FIGURE 3:**
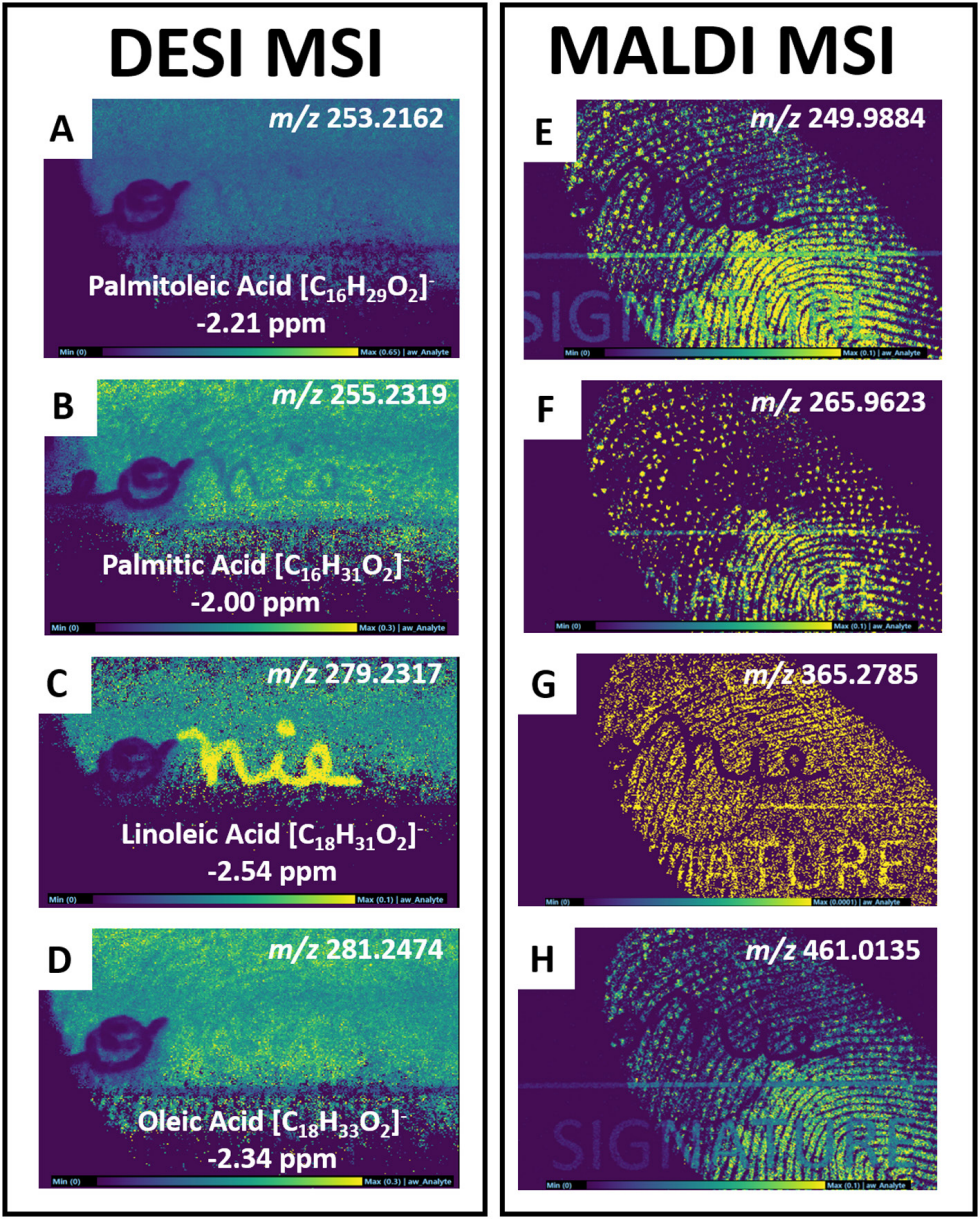
Representative fingerprint images obtained using DESI MSI (A–D) and MALDI MSI (E–H).

A schematic representation of the sample illustrates the location of the deposited fingerprints (Figure S1). The ability of DESI and MALDI MSI was compared and evaluated for their ability to visualise fingerprints in the QDE (Figure 3). The comparison was made based on factors including image clarity, and the ability to detect compounds within fingerprint residues.

Our aim was not to compare molecular identities directly, but rather to inspect ions capable of producing a successful fingerprint image. As demonstrated in Figure 3, both MSI approaches detected fingerprint‐derived molecules, indicating that each technique can provide chemical information characteristic to donor's molecular signatures. However, whilst MALDI MS images of the fingerprints range from grade 2 to grade 3 of the CAST scale [42], DESI MSI only yielded grade 1 images.

### 3.5 Fingerprint Analysis—DESI MSI

Following negative ionisation DESI MSI, endogenous lipids derived from fingerprints are detected as deprotonated fatty acids (normally present in the sebaceous and eccrine residues from sweat). More specifically, the ions observed fall within the characteristic range of C16‐C18 fatty acids with *m*/*z* 253.2162 putatively identified as palmitoleic acid (−2.21 ppm), *m*/*z* 255.2319 as palmitic acid (−2.00 ppm), *m*/*z* 279.2317 as linoleic acid (−2.54 ppm) and finally *m*/*z* 281.2474 as oleic acid (−2.34 ppm). These molecules are known to be abundant in human sebum and other skin surface lipids. The ion at *m*/*z* 279.2317, representing linoleic acid (−2.54 ppm), appears to be present within both the fingerprint region and in PEN 2 ink. This is evident by the increased signal within the PEN 2 and uniform distribution throughout the fingerprint. Notably, this ion has previously been identified in ballpoint pen inks by our group [16]. In the fingerprint, this lipid ion appeared delocalised, whereas on the pen it remained localised. The observed behaviour could be explained by differential absorption of fingerprint material into the paper substrate, while the ink layer may act as a more stable medium that helps preserve ridge detail. The delocalisation is not unique to this ion, as DESI MSI caused diffusion in all fingerprint‐related ions that appeared delocalised and distributed across the image rather than confined to ridge structures, consistent with observations reported by Krishna et al. [29].

### 3.6 Fingerprint Analysis—MALDI MSI

MALDI MSI not only allowed detection of common fingerprint‐related ions (*m*/*z* 249.9883, *m*/*z* 265.9623, *m*/*z* 365.2785 and *m*/*z* 461.0135) [27] but also generated clear ridge detail with well‐defined ridge‐furrow contrast. This enabled visualisation of fingerprint topology that was not achievable with DESI MSI under the conditions tested. Mass spectra extracted following analysis in imaging mode (specifically from the fingerprint region) can be used to infer intelligence around the suspects and/or the circumstances of the case. A representative mass spectrum extracted from the fingerprint (excluding areas overlapping with ink) is shown in Figure S5. The spectra contained several previously reported endogenous fingerprint ions, including a series of signals likely to be related to phospholipids in the *m*/*z* 650–900 range (Figure S5). More specifically, ions at *m*/*z* 672.0388, *m*/*z* 688.0131, and *m*/*z* 703.9867 have already been reported in groomed marks by Bradshaw et al. [43]. Lower mass endogenous species at *m*/*z* 249.9884, *m*/*z* 265.9624, and *m*/*z* 299.3070, previously described by Krishna et al., were also detected [27]. The chemical profile also revealed the presence of a protonated lysophospholipid (lysolecithin) and its sodium and potassium adducts. Lysolecithin is widely used in cosmetic and personal‐care formulations as a co‐emulsifier and skin‐conditioning agent [44]. Importantly, the detection of such exogenous compounds illustrates how MALDI MS analysis provides complementary chemical information that is not accessible through conventional visualisation. This additional chemical insight can support interpretative work in forensic investigations by indicating recent contact with cosmetic products, offering a deeper layer of evidence regarding a donor's activities or exposures that would otherwise remain invisible.

### 3.7 Statistical Analysis

When looking at the number of *m*/*z* detected for each feature using each analytical technique (Table 1) MALDI MSI allowed detection of a greater number of ions than DESI across most features, except for PEN 1, where DESI performed better, and PEN 2, where both techniques detected an equivalent number of ions.

The total number of detected *m*/*z* peaks was 944 for MALDI and 176 for DESI following pre‐processing and alignment. PEN 1 showed similar counts (23 vs. 21), and PEN 2 was identical (10 each). However, MALDI MSI detected substantially more peaks in printed ink (30 vs. 2), fingerprints (44 vs. 28), and paper (20 vs. 6).

## 4 Conclusion

DESI (negative mode) and MALDI (positive mode) were each operated under their optimal conditions and therefore the comparison reflects optimal performance for each technique rather than a direct polarity matched comparison. Overall, this study demonstrates that both DESI and MALDI MSI can discriminate between two different blue ballpoint pen inks through the detection of ink‐specific molecular features. MALDI MSI showed superior performance in the analysis of printed ink regions due to more efficient pigment extraction. For recovering chemical information from groomed fingerprints, both techniques successfully detected lipids, supporting their applicability for chemical profiling in forensic contexts. Importantly, MALDI MSI provided better spatial resolution, preserving ridge quality, whereas DESI MSI resulted in analyte delocalisation, which reduced ridge clarity. With respect to sample preservation and suitability for QDE, DESI MSI offers the advantage of minimal or no sample preparation, although solvent choice and geometric parameters (such as spray angle and proximity) remain critical to prevent ink dissolution and molecular spreading. DESI MSI has shown to be useful for ink analysis, however prior testing is necessary to confirm that the spray solvent does not cause ink delocalisation. Additionally, DESI MSI can be used for chemical profiling of fingerprints in scenarios where molecular information is of primary interest and high spatial fidelity (e.g., ridge detail clarity) is not required. MALDI MSI does require matrix deposition, however, the application of this technique showed minimal observable alteration to the original document, leaving the inked text clearly visible following analysis. This study further demonstrates the potential of MSI to support QDE by enabling the simultaneous analysis of ink composition and fingerprint‐associated chemical and biometric information from a single acquisition. MALDI‐MSI confirmed its suitability for fingerprint analysis and supporting its recognition by the UK Home Office and the Defence Science and Technology Laboratory (Dstl). The comparative evaluation of MALDI and DESI MSI highlights their respective strengths and limitations and provides valuable insight into their reliability and practicality for real‐world forensic document investigations. From a cost perspective, DESI MSI required fewer consumables and a shorter acquisition time (≈ 6 h), while MALDI MSI involved higher preparation and consumable costs associated with matrix application and longer acquisition time (≈ 9 h). While the present study focused on a single mock document, serving as a proof‐of‐concept, future work is necessary to evaluate the approach across multiple documents and different sample types.

## Author Contributions

**Veronika Tibljas:** methodology, software, formal analysis, investigation, data curation, writing – original draft, writing – review and editing. **Robert Bradshaw:** conceptualization, methodology, resources, writing – review and editing, supervision, project administration, funding acquisition, validation, visualization. **Simona Francese:** conceptualization, methodology, resources, writing – review and editing, supervision, project administration, validation, visualization. **Charlotte Lefebvre:** software, formal analysis, investigation, data curation, writing – review and editing. **Marjory Da Costa Abreu:** software, writing – review and editing, supervision, project administration.

## Funding

This work was supported by Sheffield Hallam University.

## Supporting information


**Data S1:** Supporting Information.

**Figure S1:** Schematic view of the simulated signed document, whereby PEN 1 was employed to write “Leo” and PEN 2 to write “nie”. A groomed fingerprint was deposited on top of the two types of inks (ballpoint and printed).

**Figure S2:** Representative example of data extraction for statistical analysis, showing the selection of ROIs (indicated by coloured squares) and the corresponding average spectra from a ROI of PEN1 (outlined in yellow) and printed ink (outlined in green).

**Figure S3:** Ions at *m/z* 395.0416, *m/z* 412.3949 and *m/z* 476.9875 (A‐C) show signal enhancement of ions in printed ink areas where fingerprint was present, whereas images of ions at *m/z* 674.0144, *m/z* 323.1474 and *m/z* 381.1093 (D‐F) show printed ink ions with signal suppression in areas where a fingerprint was deposited.

**Figure S4:** The signal intensity difference of the ion at *m/z* 470.25894, corresponding to Basic Blue 26 (−0.36 ppm) present in both PEN 1 and PEN 2.

**Figure S5:** Mass spectrum extracted from an ink‐free fingerprint. The highlighted peaks have been putatively attributed to lysolecithin: the singly charged [M + H]^+^ at *m/z* 496.3417, the sodium adduct [M + Na]^+^ at *m/z* 518.3224, and the potassium adduct [M + K]^+^ at *m/z* 534.2972.

## Data Availability

The data that supports the findings of this study are available in the Supporting Information of this article.
