# Supplementary material for: A Comparative Study of MALDI MSI Versus DESI MSI Applied to Questioned Document Examination
Source: Rapid Commun Mass Spectrom. 2026 Jul 9;40(19):e70135. doi: 10.1002/rcm.70135 (PMC13347109; doi:10.1002/rcm.70135)
Supplement: Supplementary file 1 — Data S1: Supporting Information. [file RCM-40-e70135-s002.docx]

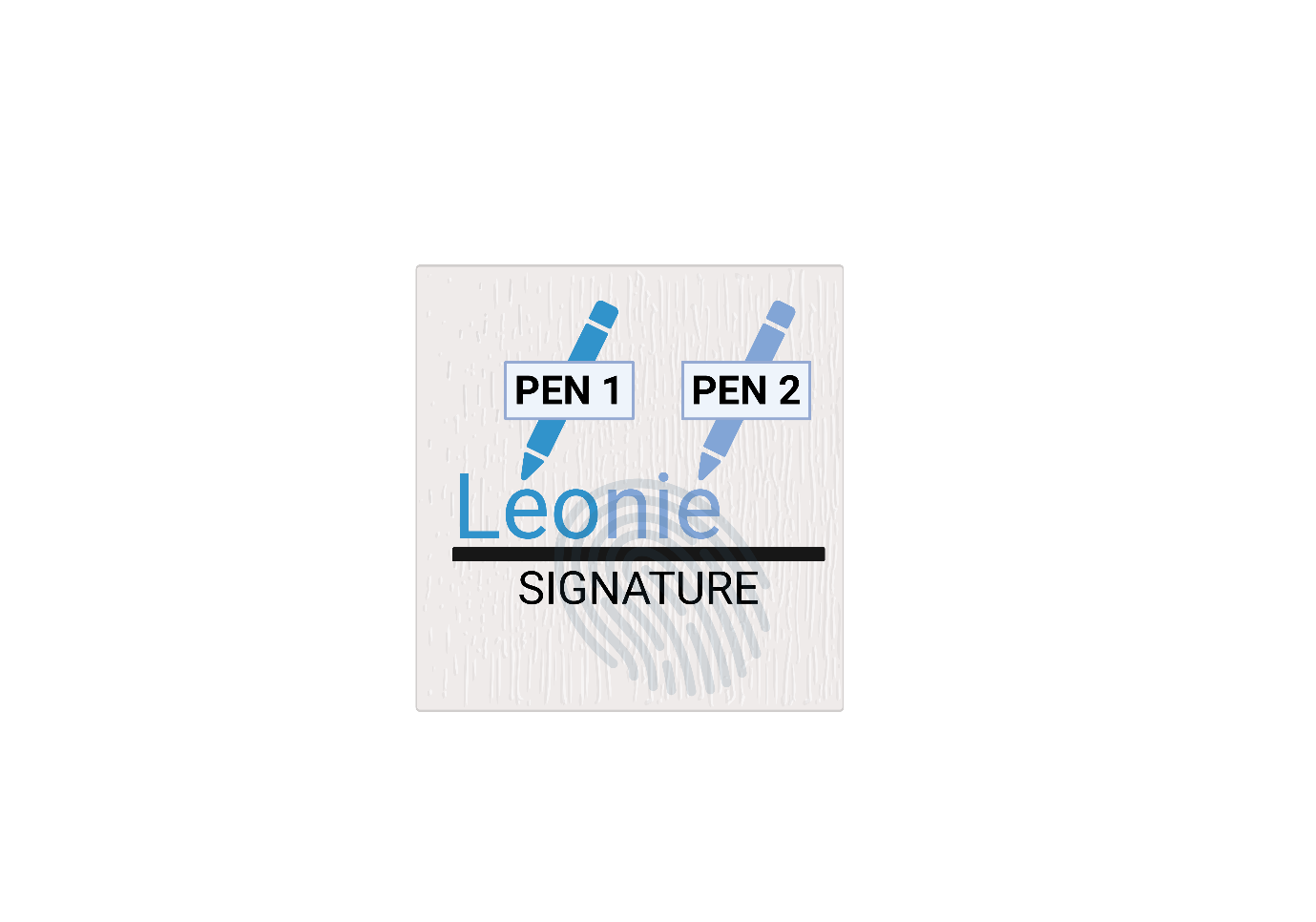


**Figure S1**- Schematic view of the simulated signed document, whereby PEN 1 was employed to write "Leo" and PEN 2 to write "nie". A groomed fingerprint was deposited on top of the two types of inks (ballpoint and printed).


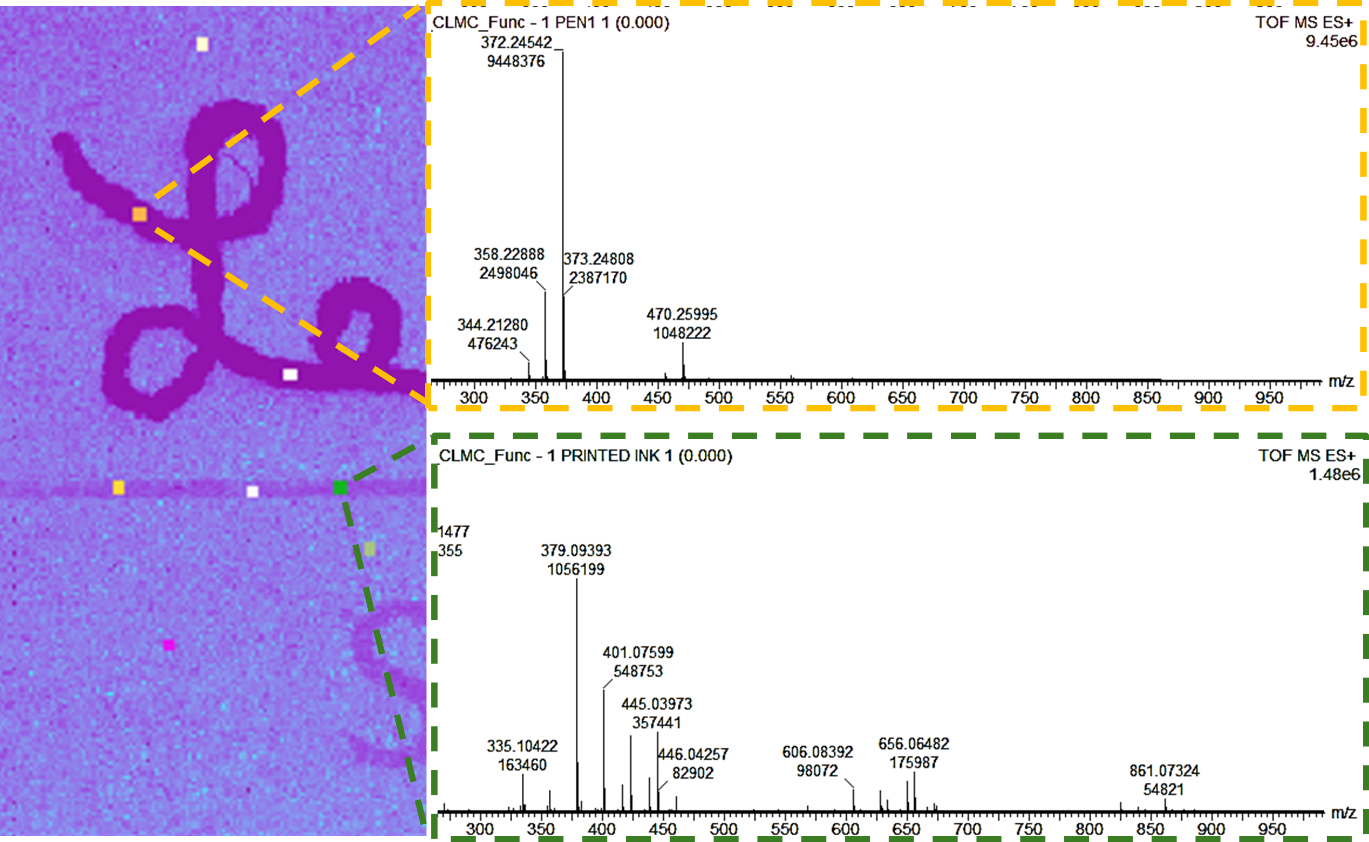


**Figure S2.** Representative example of data extraction for statistical analysis, showing the selection of ROIs (indicated by coloured squares) and the corresponding average spectra from a ROI of PEN1 (outlined in yellow) and printed ink (outlined in green).


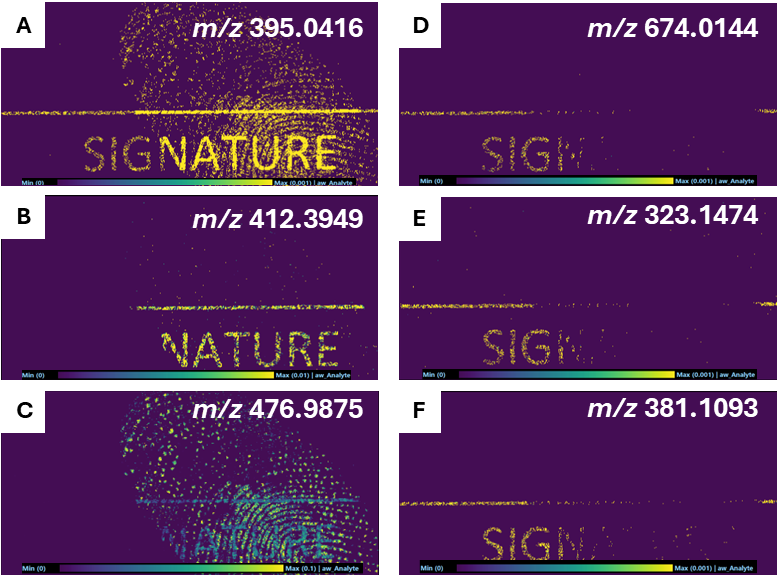


**Figure S3**. Ions at *m/z* 395.0416, *m/z* 412.3949 and *m/z* 476.9875 (A-C) show signal enhancement of ions in printed ink areas where fingerprint was present, whereas images of ions at *m/z* 674.0144, *m/z* 323.1474 and *m/z* 381.1093 (D-F) show printed ink ions with signal suppression in areas where a fingerprint was deposited.


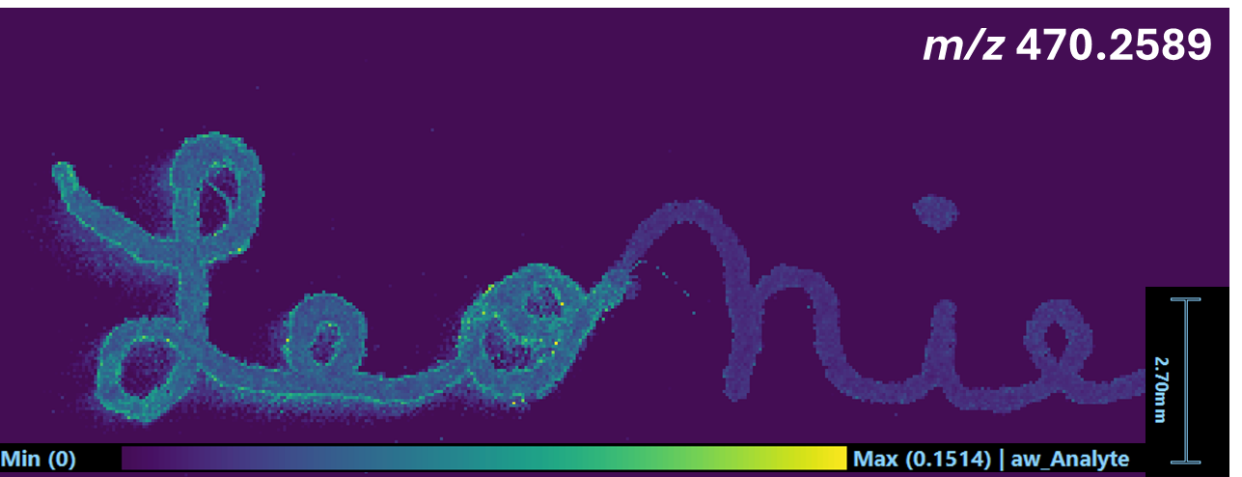


**Figure S4.** The signal intensity difference of the ion at *m/z* 470.25894, corresponding to Basic Blue 26 (-0.36 ppm) present in both PEN 1 and PEN 2.


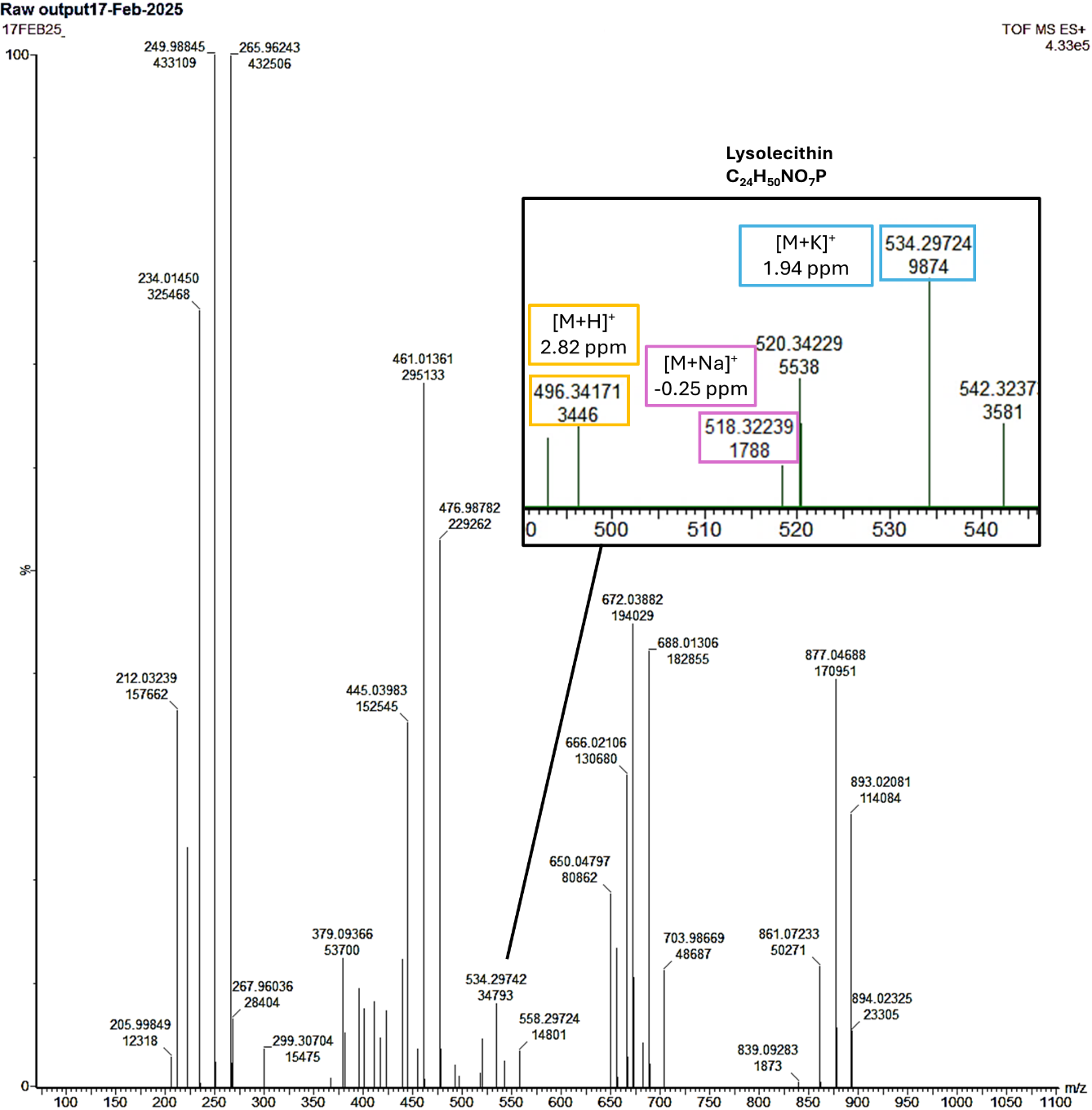


**Figure S5.** Mass spectrum extracted from an ink-free fingerprint. The highlighted peaks have been putatively attributed to lysolecithin: the singly charged [M+H]^+^ at *m/z* 496.3417, the sodium adduct [M+Na]^+^ at *m/z* 518.3224, and the potassium adduct [M+K]^+^ at *m/z* 534.2972.
