# Supplementary figures and images for: A Comparative Study of MALDI MSI Versus DESI MSI Applied to Questioned Document Examination
Source: Rapid Commun Mass Spectrom. 2026 Jul 9;40(19):e70135. doi: 10.1002/rcm.70135 (PMC13347109; doi:10.1002/rcm.70135)

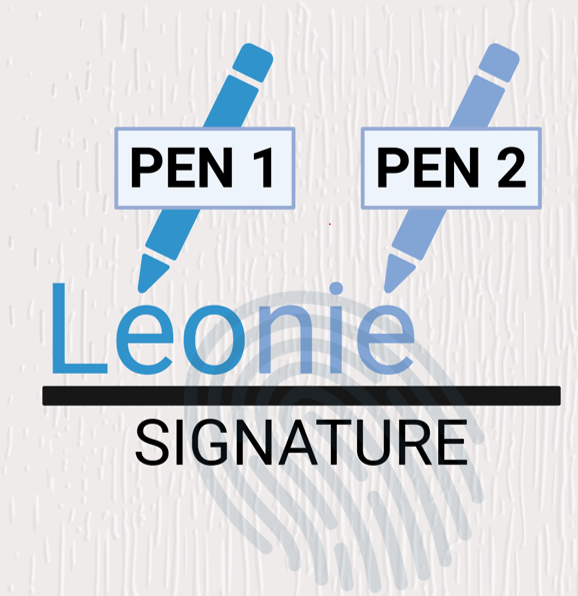

Supplement: Supplementary file 2 — Figure S1: Schematic view of the simulated signed document, whereby PEN 1 was employed to write “Leo” and PEN 2 to write “nie”. A groomed fingerprint was deposited on top of the two types of inks (ballpoint and printed). [file RCM-40-e70135-s005.tif]

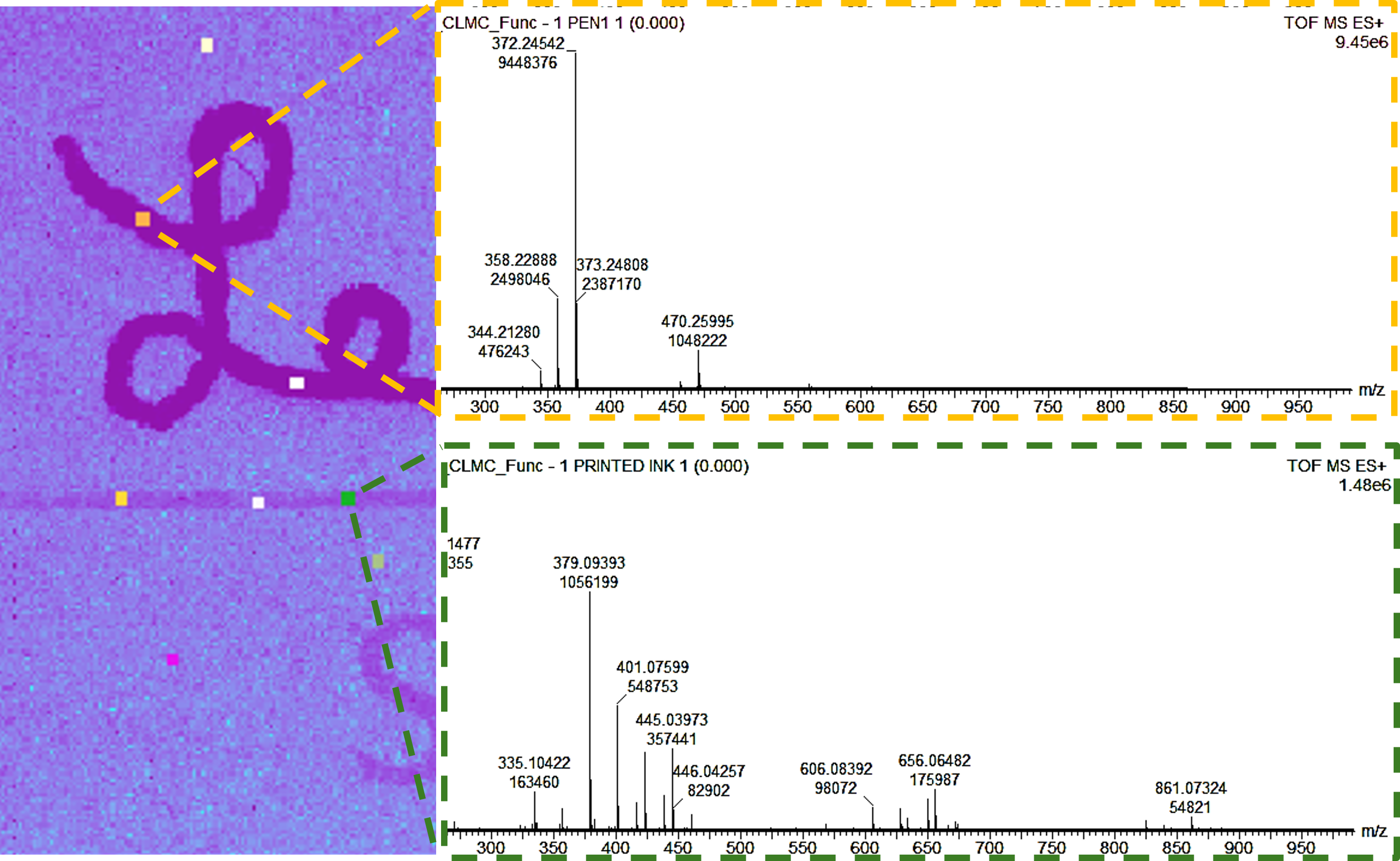

Supplement: Supplementary file 3 — Figure S2: Representative example of data extraction for statistical analysis, showing the selection of ROIs (indicated by coloured squares) and the corresponding average spectra from a ROI of PEN1 (outlined in yellow) and printed ink (outlined in green). [file RCM-40-e70135-s004.tif]

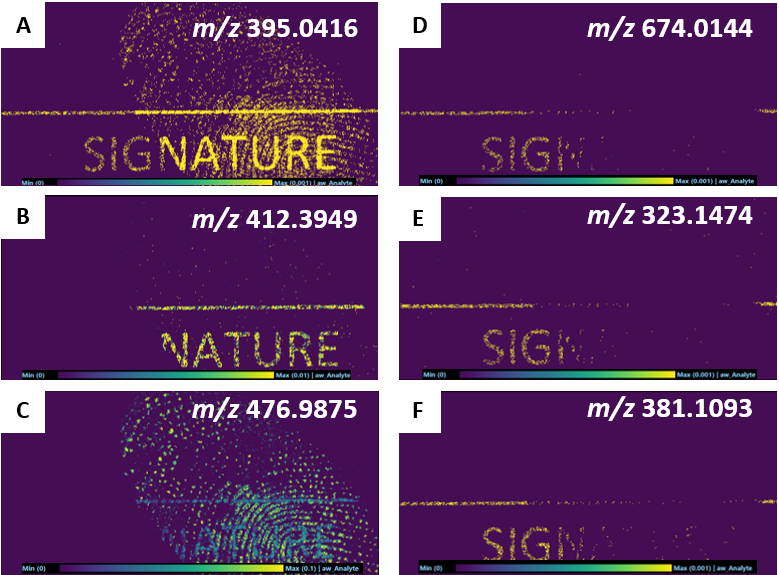

Supplement: Supplementary file 4 — Figure S3: Ions at m/z 395.0416, m/z 412.3949 and m/z 476.9875 (A‐C) show signal enhancement of ions in printed ink areas where fingerprint was present, whereas images of ions at m/z 674.0144, m/z 323.1474 and m/z 381.1093 (D‐F) show printed ink ions with signal suppression in areas where a fingerprint was deposited. [file RCM-40-e70135-s003.tif]

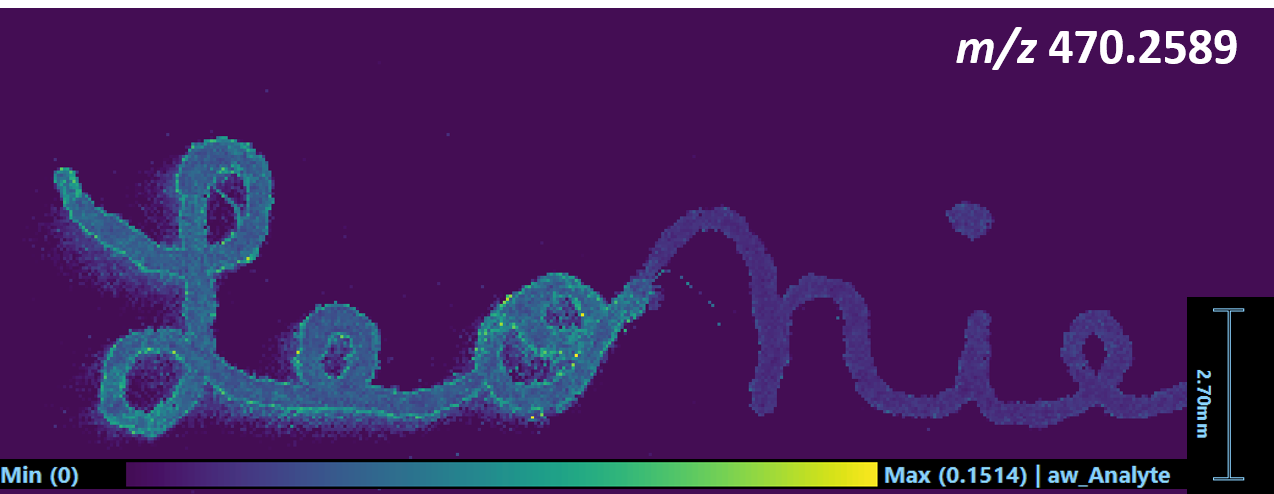

Supplement: Supplementary file 5 — Figure S4: The signal intensity difference of the ion at m/z 470.25894, corresponding to Basic Blue 26 (−0.36 ppm) present in both PEN 1 and PEN 2. [file RCM-40-e70135-s001.tif]

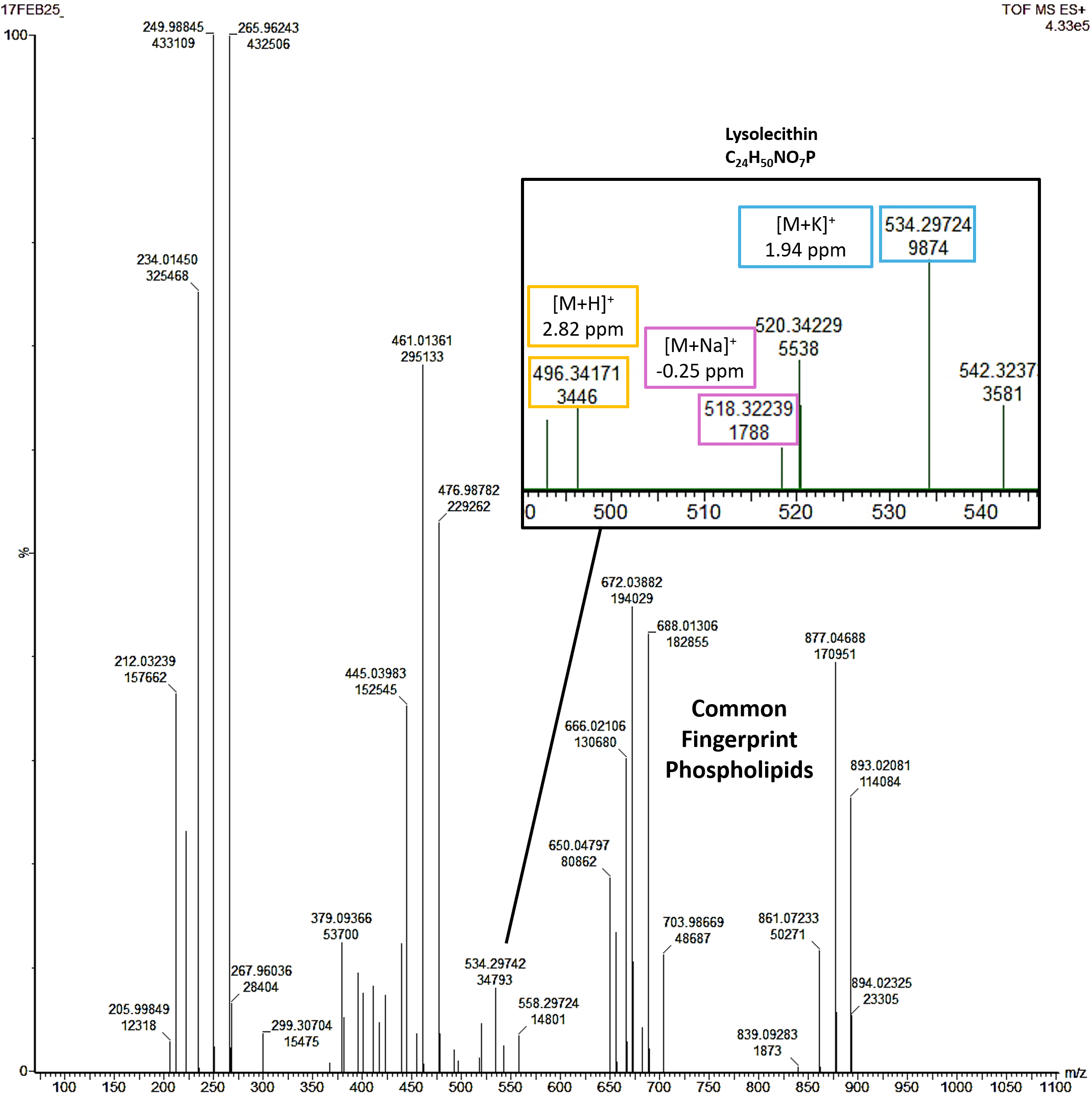

Supplement: Supplementary file 6 — Figure S5: Mass spectrum extracted from an ink‐free fingerprint. The highlighted peaks have been putatively attributed to lysolecithin: the singly charged [M + H]+ at m/z 496.3417, the sodium adduct [M + Na]+ at m/z 518.3224, and the potassium adduct [M + K]+ at m/z 534.2972. [file RCM-40-e70135-s006.tif]
